# The Association Between Online Health Information–Seeking Behaviors and Health Behaviors Among Hispanics in New York City: A Community-Based Cross-Sectional Study

**DOI:** 10.2196/jmir.4368

**Published:** 2015-11-26

**Authors:** Young Ji Lee, Bernadette Boden-Albala, Haomiao Jia, Adam Wilcox, Suzanne Bakken

**Affiliations:** ^1^ Department of Health and Community Systems School of Nursing University of Pittsburgh Pittsburgh, PA United States; ^2^ Global Institute of Public Health New York University New York, NY United States; ^3^ School of Nursing Columbia University New York, NY United States; ^4^ Intermountain Healthcare Murray, UT United States; ^5^ Department of Biomedical Informatics Columbia University New York, NY United States

**Keywords:** Internet, information-seeking behavior, health behavior, consumer health information, Hispanic Americans

## Abstract

**Background:**

Hispanics are the fastest-growing minority group in the United States and they suffer from a disproportionate burden of chronic diseases. Studies have shown that online health information has the potential to affect health behaviors and influence management of chronic disease for a significant proportion of the population, but little research has focused on Hispanics.

**Objective:**

The specific aim of this descriptive, cross-sectional study was to examine the association between online health information–seeking behaviors and health behaviors (physical activity, fruit and vegetable consumption, alcohol use, and hypertension medication adherence) among Hispanics.

**Methods:**

Data were collected from a convenience sample (N=2680) of Hispanics living in northern Manhattan by bilingual community health workers in a face-to-face interview and analyzed using linear and ordinal logistic regression. Variable selection and statistical analyses were guided by the Integrative Model of eHealth Use.

**Results:**

Only 7.38% (198/2680) of the sample reported online health information–seeking behaviors. Levels of moderate physical activity and fruit, vegetable, and alcohol consumption were low. Among individuals taking hypertension medication (n=825), adherence was reported as high by approximately one-third (30.9%, 255/825) of the sample. Controlling for demographic, situational, and literacy variables, online health information–seeking behaviors were significantly associated with fruit (β=0.35, 95% CI 0.08-0.62, *P*=.01) and vegetable (β=0.36, 95% CI 0.06-0.65, *P*=.02) consumption and physical activity (β=3.73, 95% CI 1.99-5.46, *P*<.001), but not alcohol consumption or hypertension medication adherence. In the regression models, literacy factors, which were used as control variables, were associated with 3 health behaviors: social networking site membership (used to measure one dimension of computer literacy) was associated with fruit consumption (β=0.23, 95% CI 0.05-0.42, *P*=.02), health literacy was associated with alcohol consumption (β=0.44, 95% CI 0.24-0.63, *P*<.001), and hypertension medication adherence (β=–0.32, 95% CI –0.62 to –0.03, *P*=.03). Models explained only a small amount of the variance in health behaviors.

**Conclusions:**

Given the promising, although modest, associations between online health information–seeking behaviors and some health behaviors, efforts are needed to improve Hispanics’ ability to access and understand health information and to enhance the availability of online health information that is suitable in terms of language, readability level, and cultural relevance.

##  Introduction

Hispanics are the fastest-growing minority group in the United States and they suffer from a disproportionate burden of chronic diseases compared with non-Hispanics groups [1,2]. For instance, Hispanics have similar or lower levels of hypertension compared to non-Hispanic whites; however, their high blood pressure is less likely to be treated or controlled [3,4]. To manage illnesses and associated health problems, it is especially important for people with chronic diseases to engage in health-promoting behaviors [5-7]. However, studies have shown that there are disparities in health behaviors between Hispanics and non-Hispanics; Hispanics, as a group, spend less time on healthy behaviors than other ethnic groups [8-10]. For example, Hispanics report less physical activity time than non-Hispanics and have high rates of diseases related to sedentary lifestyle [9]. Also, compared with other ethnic groups, the rise in nutrition-related chronic diseases among Hispanics reflects an unequal burden of health issues [10].

As the number of Internet users has grown, the Internet has become an important resource for chronic disease management [11]. Studies have shown that online health information has the potential to affect health behaviors and health outcomes for a significant proportion of the population [12,13]. Dutta-Bergman [14] demonstrated that online health information seekers reported higher levels of health behaviors, including physical activity, diet, alcohol consumption, medication, and smoking. In addition, online health information enables people to manage chronic diseases more effectively and improve their quality of life [15-17]. Because patient participation in health care decision making across the illness continuum is increasing, online health information–seeking behaviors are an important strategy for successful health interventions that inform decision making [18-20].

Hispanics’ interest in online health information is increasing. In 2011, the Pew Internet & American Life Project reported that 45% of Hispanic Internet users had sought health information from the Internet; this increased to 66% in 2013 [21,22]. Compared with other ethnic groups, Hispanics are more likely to seek health information from the Internet or other social media rather than from a physician because they often lack access to a regular health care provider [23,24]. However, current studies have been limited to information seeking and its influence on health behaviors among the general public. Given the growth of Hispanic online health information seekers [25,26], examining the relationships between online health information–seeking behaviors and health behaviors in Hispanics is warranted.

Our previous study focused on the correlates of online health information–seeking behaviors in Hispanics [27]. This study builds on that research by examining the association between online health information–seeking behaviors and 5 health behaviors (physical activity, fruit and vegetable consumption, alcohol use, and hypertension medication adherence) of relevance to Hispanics [2,14,28,29].

## Methods

### Study Design and Hypotheses

Data were collected during an in-depth community survey as part of the Washington Heights Inwood Informatics Infrastructure for Comparative Effectiveness Research (WICER) project. Five hypotheses were tested. Controlling for situational, sociodemographic, and literacy factors, online health information–seeking behaviors would be (1) positively associated with fruit consumption, (2) positively associated with vegetable consumption, (3) positively associated with physical activity, (4) negatively associated with alcohol consumption, and (5) positively associated with hypertension medication adherence. In addition, the associations between situational, sociodemographic, and literacy factors and fruit consumption, vegetable consumption, physical activity, alcohol consumption, and hypertension medication adherence were examined.

### Setting

The survey catchment area included 5 ZIP codes (10031, 10032, 10033, 10034, and 10040) comprising the Washington Heights Inwood community of northern Manhattan, which has been designated a medically underserved area by the Centers for Medicare and Medicaid Services because it meets the criteria related to the level of poverty, elderly, and infant mortality, and the ratio of primary care providers in the community [30,31]. Currently, 71% of Washington Heights and Inwood area residents are Hispanic and 51% of residents are immigrants [32].

### Recruitment

After approval by the Institutional Review Board of Columbia University Medical Center, recruitment of eligible participants was initiated using multiple methods. Participants were recruited from households or other places in the community (eg, schools, grocery stores, hair salons). The household sample started as a probability sample that evolved, as planned, to snowball sampling based on participants’ social networks and convenience sampling. For the probability sample, we randomized 68,000 dwelling units (ie, households) in the 5 ZIP codes and selected a weighted sample from each of the 8 New York City Department of Health and Mental Hygiene districts in the community. The sample size of each health district was weighted by the distribution of the population. Once we enrolled a household member and finished the survey, we asked participants if they would introduce the research team to members of their social network to ascertain interest in completing the survey (ie, snowball sampling). Other participants were recruited through direct contact with community health workers in community settings.

### Sample

The sample consisted of those who completed the WICER community survey between April 2012 and November 2012. Participants who were 18 years or older, English or Spanish speaking, and Hispanic were eligible to participate in this study.

### Data Collection

Data were collected via in-person interviews by bilingual community health workers in households or places in the community (eg, schools, grocery stores). For consistency of data collection across interviewers, community health workers received a combination of didactic and practical training in responsible conduct of research and specific study procedures including informed consent. Their performance was monitored by the project coordinator on a weekly basis. Before conducting the survey interview, bilingual study personnel obtained informed consent from the participant in their language of choice (English or Spanish). The process required approximately 45 to 60 minutes for completion and participants received US $25 compensation in the form of subway cards, grocery coupons, or movie tickets.

### Study Variables

After WICER survey data collection was completed, Bodie and Dutta’s Integrative Model of eHealth Use [33] was applied to inform selection of correlates and related health outcomes for online health information–seeking behaviors from among the WICER variables and to guide the data analysis. This model includes demographic information, situational factors, health literacy, computer literacy, online health information–seeking behaviors, and health behavior variables. Table 1 summarizes the definitions of model concepts and operationalization of the concepts through WICER study variables and measures. As described in more detail in study measures, only selected dimensions of health literacy and computer literacy were measured in the study (ie, the available WICER variables were more narrow than the broad concepts in the model).

**Table 1:** Conceptualization and measurement of study variables.

Concept/Definition	WICER Variable/Measure
Situational factors: the specific health situations faced by a patient and his or her subsequent consumer health information needs [34]	Hypertension: “Have you ever been told by a doctor, nurse, or other health professional that you had hypertension also called high blood pressure or pressure?” [35]
	General health status: “Would you say that in general your health is...?” [36]
	Serious health problems: “Have you experienced any serious personal health problems that have lasted for at least 6 months?” [37]
Demographic information	Age, gender, employment, immigrant status, marital status, educational level, insurance
Health literacy: the degree to which individuals have the capacity to obtain, process, and understand basic health information and services needed to make appropriate health decisions [38]	Health literacy: Chew’s 1-item health literacy screening question “How often do you have problems learning about your medical condition because of difficulty understanding written information?” [39]
Computer literacy: computer skills and ability to use technology to improve learning, productivity, and performance [40]	Social networking site membership: “Do you belong to any social networking sites like Facebook, Myspace, or Twitter?”
Online health information–seeking behaviors: the interaction of an individual with or through an electronic device or communication technology to access or transmit health information or to receive guidance and support on a health-related issue [41]	Online health information–seeking behaviors: “In the past 12 months, have you (1) participated in an online support group for people with similar health or medical issues, (2) used email or the Internet to communicate with a doctor or doctor’s office, or (3) used the Internet to look up health or medical information?” [42]
Health behaviors	Physical activity, fruit consumption, vegetable consumption
	Alcohol use: New York City Health and Nutrition Examination Survey (NYCHANES) [43]
	Medication adherence: Morisky 8-Item Medication Adherence Scale (MMAS-8) [44]

Bodie and Dutta [33] suggest that disparities in social structures, such as sociodemographic factors, lead to individual-level differences in online health information–seeking ability. This model also incorporates health literacy and computer literacy as predictors of online health information–seeking behaviors and health behaviors. In addition, it proposes that differences in health literacy are related to sociodemographic factors, such as age, race, birthplace, education level, and income, which also influence online health information–seeking behaviors [25,45]. Ultimately, this difference in online health information–seeking behaviors causes disparities in lifestyle that are related to health outcomes and continue to contribute to health care disparities [33].

#### Demographic and Situational Factors

As summarized in Table 1, situational and demographic data were obtained during the interview. For situational variables, hypertension was measured by the question “Have you ever been told by a doctor, nurse, or other health professional that you had hypertension also called high blood pressure or pressure?” [35]. The presence of serious health problems was measured by the question “Have you experienced any serious personal health problems that have lasted for at least 6 months?” from the Chronic Burden Scale [37]. Self-reported general health status was recorded in 5 categories: excellent, very good, good, fair, and poor. General health status was measured on a 5-point Likert scale (1=excellent, 5=poor) from the Short Form-8 Health Survey (SF-8) [36]. Demographic data included age, gender, employment, marital status, immigrant status, educational level, and type of insurance.

#### Health Literacy

Although the Institute of Medicine definition of health literacy is broad and encompasses accessing and processing as well as understanding and applying information, [38] we selected understanding as the focus of our health literacy measurement. To assess health literacy, we used a one-item health literacy screening measure: “How often do you have problems learning about your medical condition because of difficulty understanding written information?” Response options were always, often, sometimes, occasionally, and never. Criterion-related validity with Short Test of Functional Health Literacy in Adults (S-TOFHLA) was established by the area under the receiver operating characteristic curve (0.76) in a study of Veterans Affairs clinic patients [38]. Evidence for the reliability and validity of the 3 one-item scales from Chew et al [39] have been demonstrated in other populations, including females, African Americans [46], Turkish speakers [47], and Spanish speakers [48], This one-item scale was selected for the analysis in this paper instead of other WICER health literacy variables (eg, Newest Vital Sign which includes numeracy) because of its relevance to online health information–seeking behaviors. Moreover, Sarkar and colleagues [48] revalidated this single health literacy item among English and Spanish speakers and found similar test characteristics to previous studies; thus, it was appropriate for our primarily Spanish-speaking Hispanic participants. Per the method described by Chew and colleagues [39], the responses of always, often, and sometimes were coded as inadequate literacy; occasionally and never were coded as adequate literacy.

#### Social Networking Site Membership

There was no direct question regarding computer literacy in the WICER survey. The US Department of Education defines *computer literacy* as “computer skills and ability to use technology to improve learning, productivity, and performance” [40]. However, the definition of computer literacy changes as technology evolves [49] and several studies have included “using networked communication” as a component of computer literacy [50-52]. Use of social networking sites (SNS), whether by mobile device or desktop computer, requires a certain level of computer literacy as a foundation [53]. Therefore, based on the variables available in the WICER survey, one dichotomous question related to SNS membership was used to measure one dimension of computer literacy: “Do you belong to any social networking sites like Facebook, Myspace, or Twitter?” WICER survey variables specific to online health information–seeking behaviors also imply a level of computer literacy, but based on the Bodie and Dutta [33] model, were used to measure online health information–seeking behaviors.

#### Online Health Information–Seeking Behaviors

Robinson et al [41] defined *interactive health communication* as “the interaction of an individual—consumer, patient, caregiver, or professional—with or through an electronic device or communication technology to access or transmit health information or to receive guidance and support on a health-related issue.” Based on the definition, this study considered participation in an online support group, email communication with physicians, and using the Internet to look up health or medical information as online health information–seeking behaviors in this study. An affirmative response to at least 1 of 3 questions was coded as “yes” on online health information–seeking behaviors (no=0, yes=1).

#### Health Behaviors

Physical activity, fruit and vegetable consumption, and alcohol consumption were measured using items from New York City Health and Nutrition Examination Survey (NYCHANES) [43]. Moderate physical activity was measured by the question “Over the past 30 days, did you do moderate activities for at least 10 minutes that caused only light sweating or a slight to moderate increase in breathing or heart rate?” Answers were standardized into weekly rates. Similarly, fruit, vegetable, and dark vegetable consumption were standardized as number of servings per day. Alcohol use was first measured by the dichotomous question “Have you ever had alcoholic beverages such as beer, wine, champagne, or liquor at least once per month for 6 months or more?” If yes, the respondents were asked to select from among 9 frequencies of drinking. Responses were clustered into 3 categories: 1, 1-19, or ≥20 drinks per month [54]. Those who answered “no” to the initial question were categorized as <1.

Hypertension medication adherence was measured by the Morisky 8-Item Medication Adherence Scale (MMAS-8) [44] with respondents explicitly instructed to limit the type of medication to those taken for hypertension. Three questions were dichotomous (no=0, yes=1) and 5 questions had 4 response options: (1) never or rarely, (2) once in a while, (3) sometimes, and (4) often. The former 2 were coded as “no” and the latter 2 as “yes.” One dichotomous item, “Did you take your high blood pressure medication yesterday?” required reverse coding. The responses were summed and adherence was defined as high (0), medium (1-2), or low (>2) [44]. Internal consistency reliability was good (α=.75) for the study sample.

### Statistical Analysis

Data were analyzed using SPSS version 20.0 software. Univariate analyses were used to examine the frequency and distribution of study variables, calculating mean and standard deviation, range, frequency, and percentage as appropriate. The statistical analyses were guided by Bodie and Dutta’s Integrative Model of eHealth Use [33]. First, the relationships between demographics, situational and literacy (health literacy, SNS membership) variables, and online health information–seeking behaviors were examined using chi-square tests, *t* tests, and binary logistic regression with online health information–seeking behavior as the dependent variable. Although we previously conducted analyses of the variables associated with online health information–seeking behaviors in the larger WICER sample [27], analysis in this smaller sample was required to determine which variables should be controlled for in the hypothesis testing. Second, to test the study hypotheses, variables found to be significant in online health information–seeking behavior analyses were controlled for in regression models that examined the influence of online health information–seeking behaviors on health behaviors. Linear regression was used for continuous health behavior variables (physical activity and fruit and vegetable consumption). Ordinal regression models were used for alcohol use and hypertension medication adherence because those were coded as ordered categorical data. For each health behavior regression, 3 models were run: (1) demographic and situational variables only, (2) demographic and situational variables plus literacy variables, and (3) demographic, situational, and literacy variables plus online health information–seeking behaviors. The level of significance for testing of each model was set to an alpha of .05.

## Results

### Characteristics of Respondents

#### Sample Characteristics

The characteristics of the sample (N=2680) are summarized in Table 2. The mean age was 50.0 years (SD 17.1, range 18-100). Most were female (71.60%, 1919/2680) and immigrants (87.65%, 2349/2680). More than half of the respondents were unemployed (63.17%, 1693/2680), not married (64.33%, 1724/2680), and approximately half of the respondents had less than high school education (49.81%, 1335/2680). In all, 75.82% (2032/2680) of the participants were Medicare or Medicaid beneficiaries. Most respondents reported that their general health status as at least “good” (74.40%, 1994/2680) and that they did not have serious health problems (92.20%, 2471/2680). A total of 36.79% (986/2680) of respondents answered that they had been diagnosed with hypertension. More than the half of respondents (57.95%, 1553/2680) had inadequate health literacy and only 22.20% (595/2680) of respondents answered that they were a SNS member. Regarding online health information–seeking behaviors, only 7.38% (198/2680) of respondents reported that they had sought health information through the Internet. This was 29.0% (198/682) of the 682 respondents who reported using the Internet.

#### Health Behaviors

Respondents reported consuming fruit (mean 0.8, SD 1.5) and vegetables (mean 0.8, SD 1.7) less than once per day. The mean frequency of moderate physical activity was 1.6 times per week (SD 11.4). More than half of respondents (69.07%, 185/2680) reported consuming less than one alcoholic drink per month and a small percentage (2.0%, 54/2680) answered that they consumed more than 20 alcoholic drinks per month. Among individuals taking hypertension medication (n=825), adherence was reported as high (30.9%, 255/825), medium (26.3%, 217/825), and low (42.8%, 353/825).

### Relationships Between Demographic, Situational, and Literacy Variables and Online Health Information–Seeking Behaviors

In bivariate analyses related to demographic and situational variables and respondents’ online health information–seeking behaviors, there were statistically significant differences in age (*P*=.001), education (*P*<.001), employment status (*P*<.001), hypertension (*P*<.001), insurance (*P*=.005), immigrant status (*P*<.001), general health status (*P*<.001), health literacy (*P*<.001), and SNS membership (*P*<.001). Other demographic and situational variables were also significant in the binomial logistic regressions: older age (OR 1.68, 95% CI 1.29-2.20, *P*<.001), a high level of education (OR 3.07, 95% CI 1.99-4.80, *P*<.001), being born in the United States (OR 1.68, 95% CI 1.10-2.56, *P*=.02), worse health status (OR 0.39, 95% CI 0.27-0.57, *P*<.001), and lack of hypertension (OR 0.64, 95% CI 0.42-0.99, *P*=.047) were associated with participants’ online health information–seeking behaviors. Controlling for these variables, respondents with adequate health literacy (OR 2.13, 95% CI 1.52-2.99, *P*<.001) and SNS members (OR 4.21, 95% CI 2.86-6.19, *P*<.001) had increased odds of online health information–seeking behaviors.

### Hypotheses Testing: Relationships Between Online Health Information–Seeking Behaviors and Health Behaviors Controlling for Demographic, Situational, and Literacy Factors

Models 1 and 2 in Tables 3-7 are displayed to reflect the relationships between the final regressions and the eHealth use framework that guided the analysis. Model 1 shows the results of the demographic and situational variables regressed on the health behaviors and Model 2 adds the literacy variables. In terms of hypothesis testing, controlling for demographic, situational, and literacy factors (as shown in Model 3 in Tables 3-7), online health information–seeking behaviors were positively associated with fruit consumption (β=0.35, 95% CI 0.08-0.62, *P*=.01; Table 3), vegetable consumption (β=0.36, 95% CI 0.06-0.65, *P*=.02; Table 4), and physical activity (β=3.73, 95% CI 1.99-5.46, *P*<.001; Table 5). Thus, hypotheses 1-3 were supported. Other variables significantly related with health behaviors in Model 3 were age (β=–0.12, 95% CI –0.23 to –0.01, *P*=.04), immigrant status (β=–0.33, 95% CI –0.56 to –0.10, *P*=.01), general health status (β=–0.24, 95% CI –0.41 to –0.08, *P*=.01), and SNS membership (β=0.23, 95% CI 0.05-0.42, *P*=.02) for fruit consumption; immigrant status for vegetable consumption (β=–0.33, 95% CI –0.59 to –0.08, *P*=.01); and age (β=–0.87, 95% CI –1.60 to –0.13, *P*=.02), general health status (β=–1.92, 95% CI –3.00 to –0.83, *P*=.001), and education level (β=1.28, 95% CI 0.29-2.27, *P*=.01) for physical activity. However, each model explained only a small amount of the variance in each health behavior.

**Table 2:** Descriptive characteristics of Hispanic participants (N=2680).

Variables			Respondents
**Demographic factors**			
	**Gender, n (%)**		
		Men	747 (27.87)
		Women	1919 (71.60)
	**Employment status, n (%)**		
		Employed	987 (36.83)
		Unemployed	1693 (63.17)
	**Marital status, n (%)**		
		Married/living as	935 (34.89)
		Otherwise	1724 (64.33)
	**Education, n (%)**		
		<High school graduate	1335 (49.81)
		≥High school graduate	1298 (48.43)
	**Birthplace, n (%)**		
		Born in the United States	323 (12.05)
		Born in the other countries	2349 (87.65)
	**Insurance, n (%)**		
		Medicare/Medicaid	2032 (75.82)
		Others (VA, private, etc)	392 (14.63)
		None	356 (13.28)
	Age (years), mean (SD)		50.0 (17.1)
**Situational factors**			
	**General health status, n (%)**		
		<Good	612 (22.84)
		≥Good	1994 (74.40)
	**Hypertension, n (%)**		
		Yes	986 (36.79)
		No	1662 (62.01)
	**Serious personal health problems, n (%)**		
		Yes	187 (6.98)
		No	2471 (92.20)
**Literacy factors**			
	**Health literacy, n (%)**		
		Adequate literacy	1009 (37.65)
		Inadequate literacy	1553 (57.95)
	**SNS membership, n (%)**		
		Yes	595 (22.20)
		No	1750 (65.30)
	**Online health information–seeking behaviors, n (%)**		
		Yes	198 (7.39)
		No	2471 (92.20)
**Health behaviors**			
	Physical activity (times/week), mean (SD)		1.6 (11.4)
	Fruit consumption (times/day), mean (SD)		0.8 (1.5)
	Vegetable consumption (times/day), mean (SD)		0.8 (1.7)
	**Alcohol consumption, n (%)**		
		<1 per month	1851 (69.07)
		1-19 per month	660 (24.63)
		≥20 per month	54 (2.01)
	**Medication adherence,** ^a^ **n (%)**		
		High adherence	255 (30.9)
		Medium adherence	217 (26.3)
		Low adherence	353 (42.8)

^a^ Sample size for medication adherence is n=825.

**Table 3:** Linear regression: association between online health information–seeking behaviors and fruit consumption.

Variables		Model 1^a^			Model 2^b^			Model 3^b^		
		β (SE)	95% CI	*P*	β (SE)	95% CI	*P*	β (SE)	95% CI	*P*
**Demographic and situational factors**										
	Immigrant status^c^	–0.24 (0.12)	–0.47, –0.01	.04	–0.31 (0.12)	–0.54, –0.08	.01	–0.33 (0.12)	–0.56, –0.09	.01
	Age	–0.14 (0.06)	–0.26, –0.03	.01	–0.11 (0.06)	–0.22, 0.01	.07	–0.12 (0.06)	–0.24, –0.01	.04
	General health status	–0.27 (0.09)	–0.44, –0.10	.002	–0.27 (0.09)	–0.44, –0.10	.002	–0.24 (0.09)	–0.41, –0.08	.01
	Hypertension^d^	–0.01 (0.08)	–0.17, 0.15	.92	0.02 (0.08)	–0.14, 0.18	.81	0.03 (0.08)	–0.13, 0.19	.70
	Education level	0.13 (0.08)	–0.03, 0.28	.10	0.08 (0.08)	–0.08, 0.23	.32	0.05 (0.08)	–0.10, 0.21	.50
**Literacy factors**										
	Health literacy				–0.03 (0.07)	–0.15, 0.14	.97	–0.02 (0.07)	–0.17, 0.12	.76
	SNS membership^d^				0.28 (0.09)	0.10, 0.47	.01	0.23 (0.10)	0.05, 0.42	.02
Online health information–seeking behaviors^d^								0.35 (0.14)	0.08, 0.62	.01

^a^ Adjusted *R* ^*2*^=.01, *P*=.001.

^b^ Adjusted *R* ^*2*^=.01, *P*<.001.

^c^ Birthplace was coded as 0=foreign born or 1=US born.

^d^ Coded as 0=no or 1=yes.

**Table 4:** Linear regression: association between online health information–seeking behaviors and vegetable consumption.

Variables		Model 1^a^			Model 2^b^			Model 3^c^		
		β (SE)	95% CI	*P*	β (SE)	95% CI	*P*	β (SE)	95% CI	*P*
**Demographic and situational factors**										
	Immigrant status^c^	–0.28 (0.13)	–0.53, –0.02	.03	–0.31 (0.13)	–0.57, –0.06	.02	–0.33 (0.13)	–0.59, –0.08	.01
	Age	–0.10 (0.06)	–0.22, 0.02	.11	–0.07 (0.06)	–0.19, 0.06	.28	–0.09 (0.06)	–0.21, 0.04	.19
	General health status	–0.09 (0.09)	–0.28, 0.09	.31	–0.11 (0.09)	–0.29, 0.08	.26	–0.08 (0.09)	–0.26, 0.11	.41
	Hypertension^d^	–0.04 (0.09)	–0.21, 0.14	.68	–0.02 (0.09)	–0.19, 0.16	.85	–0.01 (0.09)	–0.18, 0.17	.95
	Education level	0.11 (0.08)	–0.06, 0.27	.21	0.07 (0.09)	–0.10, 0.24	.40	0.05 (0.09)	–0.12, 0.22	.60
**Literacy factors**										
	Health literacy				–0.12 (0.08)	–0.28, 0.04	.14	–0.14 (0.08)	–0.30, 0.02	.08
	SNS membership^d^				0.20 (0.10)	–0.001, 0.40	.05	0.15 (0.11)	–0.06, 0.36	.15
Online health information–seeking behaviors^d^								0.36 (0.15)	0.06, 0.65	.02

^a^ Adjusted *R* ^*2*^=.002, *P*=.12.

^b^ Adjusted *R* ^*2*^=.004, *P*=04.

^c^ Adjusted *R* ^*2*^=.006, *P*=.009.

^c^ Immigrant status was coded as 0=foreign born or 1=US born.

^d^ Coded as 0=no or 1=yes.

**Table 5:** Linear regression: association between online health information–seeking behaviors and physical activity.

Variables		Model 1^a^			Model 2^a^			Model 3^b^		
		β (SE)	95% CI	*P*	β (SE)	95% CI	*P*	β (SE)	95% CI	*P*
**Demographic and situational factors**										
	Immigrant status^c^	–0.90 (0.75)	–2.36, 0.57	.23	–1.19 (0.76)	–2.68, 0.30	.12	–1.40 (0.76)	–2.89, 0.08	.06
	Age	–0.83 (0.37)	–1.54, –0.11	.02	–0.70 (0.38)	–1.43, 0.04	.002	–0.87 (0.38)	–1.60, –0.13	.02
	General health status	–2.30 (0.55)	–3.37, –1.21	<.001	–2.22 (0.56)	–3.23, –1.12	<.001	–1.92 (0.55)	–3.00, –0.83	.001
	Hypertension^d^	0.47 (0.52)	–0.56, 1.45	.37	0.58 (0.53)	–0.45, 1.61	.27	0.71 (0.53)	–0.32, 1.74	.18
	Education level	1.75 (0.50)	0.78, 2.72	<.001	1.55 (0.50)	0.56, 2.54	.002	1.28 (0.51)	0.29, 2.27	.01
**Literacy factors**										
	Health literacy				0.99 (0.47) ^a^	0.07, 1.91	.04	0.77 (0.47)	–0.1, 1.70	.10
	SNS membership^d^				1.16 (0.60)	–0.02, 2.34	.05	0.67 (0.61)	–0.53, 1.86	.28
Online health information–seeking behaviors^d^								3.73 (0.88)	1.99, 5.46	<.001

^a^ Adjusted *R* ^*2*^=.01, *P*<.001.

^b^ Adjusted *R* ^*2*^=.02, *P*<.001.

^c^ Immigrant status was coded as 0=foreign born or 1=US born.

^d^ Coded as 0=no or 1=yes.

The hypotheses related to the association between online health information–seeking behaviors and alcohol consumption (Table 6) and hypertension medication adherence (Table 7) were not supported. For other variables in Model 3, health literacy (β=0.44, 95% CI 0.24-0.63, *P*<.001) was significantly associated with alcohol consumption and the final model explained 2.0% of the variance in alcohol consumption (*R* ^*2*^=.02, *P*<.001). Age (β=–0.27, 95% CI –0.51 to –0.02, *P*=.04), education level (β=–0.58, 95% CI –0.90 to –0.25, *P*<.001), and health literacy (β=–0.32, 95% CI –0.62 to –0.03, *P*=.03) were significantly associated with hypertension medication adherence and Model 3 explained 5.0% of the variance (*R* ^*2*^=.05, *P*<.001).

**Table 6:** Ordinal logistic regression: association between online health information–seeking behaviors and alcohol consumption.

Variables		Model 1^a^			Model 2^b^			Model 3^b^		
		β (SE)	95% CI	*P*	β (SE)	95% CI	*P*	β (SE)	95% CI	*P*
**Demographic and situational factors**										
	Immigrant status^c^	0.24 (0.14)	–0.04, 0.52	.10	0.25 (0.16)	–0.05, 0.55	.11	0.23 (0.16)	–0.07, 0.54	.13
	Age	0.07 (0.07)	–0.07, 0.21	.33	0.13 (0.08)	–0.03, 0.28	.10	0.11 (0.08)	–0.04, 0.27	.16
	General health status	0.24 (0.11)	0.02, 0.46	.03	0.20 (0.12)	–0.03, 0.43	.09	0.21 (0.12)	0.02, 0.45	.08
	Hypertension^d^	–0.03 (0.10)	–0.24, 0.17	.75	–0.03 (0.11)	–0.25, 0.19	.78	–0.02 (0.11)	–0.24, 0.20	.87
	Education level	0.08 (0.10)	–0.11, 0.27	.39	0.05 (0.11)	–0.16, 0.26	.61	0.04 (0.11)	–0.17, 0.26	.68
**Literacy factors**										
	Health literacy				0.46 (0.10)	0.26, 0.65	<.001	0.44 (0.10) ^c^	0.24, 0.63	.<001
	SNS membership^d^				0.17 (0.13)	–0.08, 0.41	.19	0.13 (0.13) ^c^	–0.12, 0.39	.29
Online health information–seeking behaviors^d^								–0.17 (0.18)	–0.52, 0.19	.35

^a^ Adjusted *R* ^*2*^=.01, *P*=.07 .

^b^ Adjusted *R* ^*2*^=.02, *P*<.001.

^c^ Immigrant status was coded as 0=foreign born or 1=US born.

^d^ Coded as 0=no or 1=yes.

**Table 7:** Ordinal logistic regression: association between online health information–seeking behaviors and hypertension medication adherence (n=825).

Variables		Model 1^a^			Model 2^b^			Model 3^c^		
		β (SE)	95% CI	*P*	β (SE)	95% CI	*P*	β (SE)	95% CI	*P*
**Demographic and situational factors**										
	Immigrant status^d^	1.31 (0.53)	0.27, 2.34	.01	0.89 (0.57)	–0.23, 2.00	.15	0.90 (0.57)	–0.22, 2.02	.12
	Age	–0.29 (0.12)	–0.51,–0.06	.01	–0.28 (0.13)	–0.52, –0.03	.03	–0.27 (0.13)	–0.51, –0.02	.04
	General health status^e^	0.33 (0.14)	0.05, 0.61	.02	0.22 (0.15)	–0.08, 0.52	.15	0.24 (0.16)	–0.01, 0.54	.13
	Education level	–0.40 (0.15)	–0.69,–0.11	.01	–0.55 (0.16)	–0.87, –0.23	.001	–0.58 (0.17) ^c^	–0.90, –0.25	<.001
**Literacy factors**										
	Health literacy				–0.32 (0.15)	–0.61,–0.03	.03	–0.32 (0.15)	–0.62, –0.03	.03
	SNS membership^e^				0.44 (0.29)	–0.14, –1.00	.13	0.41 (0.29)	–0.17, 0.98	.17
Online health information–seeking behaviors^e^								–0.64 (0.40)	–1.42, 0.14	.11

^a^ Adjusted *R* ^*2*^=.03, *P*<.001.

^b^ Adjusted *R* ^*2*^=.04, *P*<.001.

^c^ Adjusted *R* ^*2*^=.05, *P*=.

^d^ Immigrant status was coded as 0=foreign born or 1=US born.

^e^ Coded as 0=no or 1=yes.

## Discussion

### Principal Results

Only 7.38% (198/2680) of respondents reported that they had sought health information through the Internet; this was 29.0% (198/682) of the 682 respondents who indicated that they used the Internet. This is significantly different than the 66% of Hispanic Internet users who reported online health information–seeking behaviors according to the Pew Internet & American Life Project in 2013 [21]. The difference may be a reflection of the low socioeconomic status of the Washington Heights and Inwood area in this study that may have limited participants’ ability to pay for Internet access. Alternatively, we did not specifically ask about accessing the Internet via cellular telephone and respondents may have failed to consider this mode of access in their response. Thus, the actual rate of online health information–seeking behaviors in our sample may be higher than we captured in the WICER survey.

Guided by Bodie and Dutta’s Integrative Model of eHealth Use [33], demographic, situational, and literacy factors were examined to determine which variables were significantly correlated with online health information–seeking behaviors as a preliminary step to determine which variables to control for in hypothesis testing of the relationship between online health information–seeking behaviors and health behaviors. Significant demographic variables associated with online health information–seeking behaviors were older age, higher level of education, and being born in the United States. Education level and immigrant status were consistent with previous studies [55-58]; however, the relationship of age to online health information–seeking behaviors contrasted with existing studies [55-57]. This may be explained by the increasing interest in online health information among older adults. More than half of our sample was older than age 40 years (1850/2680, 69.03%), and 19.70% (528/2680) were older than age 65 years. Consistent with our prior analysis using the larger WICER sample [27], worse health status and lack of hypertension were associated with participants’ online health information–seeking behaviors.

Previous research has provided evidence that online health information seekers tend to improve their health behaviors [59-61]. Controlling for demographic, situational, and literacy factors, this study tested 5 hypotheses about the association between Hispanics’ online health information–seeking behaviors and health behaviors: fruit consumption, vegetable consumption, physical activity, alcohol consumption, and hypertension medication adherence. Although only a small proportion of the sample reported online health information–seeking behaviors, online health information–seeking behaviors were positively associated with fruit and vegetable consumption and physical activity. Our study showed that online health information seekers were likely to consume more fruits and vegetables and report more physical activity than nonseekers. However, the average fruit and vegetable consumption and physical activity levels were significantly less than the Centers for Disease Control and Prevention guidelines [62]. Moreover, the models explained only a small portion of the variance. Consequently, these findings, although promising, must be interpreted cautiously.

In contrast, online health information–seeking behaviors were not significantly associated with alcohol consumption or adherence to hypertension medication, which is inconsistent with previous studies [63-66]. There are several possible reasons for this. First, other studies did not include a large number of immigrant Hispanics and findings may vary according to these demographic characteristics. Second, there may be lack of online content on medication adherence and alcohol consumption targeted for Hispanics, who were the focus of our study, and that could limit the motivation to seek such information online. Third, a study by Shabab et al [66] suggested that online health information may not be equally effective for all types of health behaviors, which may shed light on why our findings differed for various health behaviors.

In addition to the hypothesis testing, the study revealed relationships between the demographic, situational, and literacy factors, which served as control variables in the regressions related to the 5 health behaviors. There were significant relationships between health behaviors and the demographic characteristics of age (fruit and vegetable consumption, medication adherence), education level (physical activity, hypertension medication adherence), and immigrant status (fruit and vegetable consumption). In terms of immigrant status, consistent with previous research [67,68], foreign-born Hispanic participants were likely to eat more fruit and vegetables than US-born participants. Our results showed that younger people were likely to eat more fruit and have higher physical activity levels than older people, which contrasts with existing literature [69-71]. Our findings also showed that individuals with higher education were more likely to engage in physical activity and more likely to adhere to hypertension medication regimens. In regards to the latter, findings related to education level and age were inconsistent with existing literature [72,73] that showed that lower educational attainment and older age were positively associated with medication adherence. One reason for the differences may be that our study specifically focused on hypertension medication adherence rather than medication adherence in general.

In terms of situational factors, poor health status was associated with more fruit consumption and physical activity. This is consistent within Shim’s [74] finding that people who perceived their health status as low were more likely to change their behavior after searching for health information online. Although a previous study showed having hypertension was significantly associated with health behaviors such as alcohol consumption and physical activities [75], our study did not show a significant association between hypertension and health behaviors.

Additionally, literacy factors were associated with some health behaviors: SNS membership was associated with fruit consumption and health literacy was associated with alcohol consumption and medication adherence. Previous research has reported that study participants who frequently visit SNS were willing to communicate with others about health-related issues and exchange health information through those sites [76-78]. A large survey based on 23,000 people in the United States indicated that 41% of respondents had used any social media as a health information resource; 94% of those users chose Facebook to seek health-related information [77]. Consequently, one explanation for the association between SNS membership and health behaviors is that SNS members may use those sites to find or discuss health information related to healthy diet, such as eating fruit. SNS have the potential be used as a channel to convey health information to promote health behavior, especially tailored to SNS members through user profiles, forums, blogs, comments, search queries, and tags [79].

Moreover, our study findings that participants with higher health literacy were more likely to consume less alcohol [80-82] and to adhere to hypertension medication were consistent with previous studies [83,84]. Differences in health literacy are often related to sociodemographic factors that also influence health behaviors [33,45]. Hispanics are an important target in efforts to improve health literacy due to their lower health literacy levels when compared to other ethnic groups [85]. Adequate health literacy is a prerequisite to directly influence health behaviors and to affect online health information–seeking behavior as a strategy for improving health behaviors [86]. In fact, although we conceptualized it separately in our study, effective health information–seeking behavior is one dimension of the Institute of Medicine definition of health literacy [38].

The significant associations in our study between online health information–seeking behaviors and some health behaviors as well as between our measures of literacy factors (health, SNS membership) and fruit consumption, alcohol consumption, and hypertension suggest that strategies are needed in 2 areas to promote use of online health information by Hispanics. First, skills related to health literacy and online health information–seeking behaviors must be strengthened. Finding health information online challenges those with poor health literacy and limited skills related to online health information–seeking behaviors [87-89] and most existing search tools require high levels of computer literacy for optimal use [90,91]. Second, understandable online health information is necessary to fulfill the information needs of Hispanics so that online health information is perceived as useful [92-94]. More than half of our sample had difficulty understanding written information related to their medical problems. Moreover, online health information is rarely provided in Spanish [95] and Spanish online health information is often either at an advanced reading level or of poor quality [88]. Several authors suggest that the reading level for online information should be at the grade 7 or 8 level [93,94], but that may be too high for those with low health literacy. Additionally, although not explicitly examined in this study because all participants were Hispanic and predominantly from the Dominican Republic, experts recommend that the cultural beliefs and values of the designated population also should be reflected in the content of online health information to encourage ease of use and health behavior change [96,97]. Our study may provide a foundation to understand the characteristics of Hispanics living in the Washington Heights and Inwood community, which may support the dissemination of online information more strategically [11].

### Limitations

This study has several potential limitations. First, the generalizability is limited due to the nonprobability sample and the fact that the Hispanics in our sample were primarily from the Dominican Republic; our sample does not reflect the heterogeneity of socioeconomic status, culture, and health of Hispanics in the United States [11,34,98,99]. Second, the ratio of Internet users who reported online health information–seeking behaviors may be artificially high; the number of Internet users is most likely underestimated because it was not directly asked in the WICER survey. Rather, it was calculated from the unique number of respondents who indicated either online health information–seeking behaviors or SNS use. Third, the data were cross-sectional rather than longitudinal, thus it is not possible to draw causal inferences between online health information–seeking behaviors and health behaviors. Fourth, because all measures were self-reported, recall bias and social desirability bias [100] may have influenced participants’ responses. Fifth, our measures of health literacy and computer literacy were narrower than the broader definitions in the conceptual model used for selection and analysis of variables. In particular, SNS membership alone in the absence of measurements of use may not accurately reflect overall level of computer literacy. Sixth, the survey was conducted by face-to-face interview by multiple community health workers. Although the interviewers were carefully trained and monitored, differences among interviewers may have affected the survey result [101]. However, face-to-face survey administration by bilingual community health workers was critical to the success of the WICER study given the documented underrepresentation of Hispanics in research and the low levels of health literacy in the study sample. Lastly, the questions related to online health information–seeking behaviors did not explicitly query the use of cellular phones and participants may not have considered this avenue of accessing the Internet in their response. This may be particularly important given that Hispanics are more likely to access the Internet from mobile phones than from desktop computers [21]. For example, the Pew Hispanic Center reported that Hispanics are more likely to access the Internet through mobile devices compared to non-Hispanics; 76% of Hispanics access the mobile Internet compared to 60% of non-Hispanics [102]. Because we only asked participants about the use of “Internet” there is the possibility that participants did not count the use of mobile Internet as the use of Internet. Therefore, this study may have underestimated online health information–seeking behaviors among the survey respondents.

### Conclusions

To our knowledge, this is the first large-scale study to examine the relationship between online health information–seeking behaviors and health behaviors in the Hispanic population. Although conclusions from a single study should be interpreted carefully, the data in this study suggest potential avenues for informatics-based health interventions. Given the promising, although modest, associations between online health information–seeking behaviors and 3 health behaviors (fruit and vegetable consumption and physical activity) efforts are needed to improve Hispanics’ ability to access and understand health information and to enhance the availability of online health information that is suitable in terms of language, readability level, and culture.

## Abbreviations

MMAS-8: Morisky 8-Item Medication Adherence Scale
NYCHANES: New York City Health and Nutrition Examination Survey
SNS: social networking site
WICER: Washington Heights Inwood Informatics Infrastructure for Comparative Effectiveness Research
